# Glucocorticoids’ treatment impairs the medium-term immunogenic response to SARS-CoV-2 mRNA vaccines in Systemic Lupus Erythematosus patients

**DOI:** 10.1038/s41598-022-18996-x

**Published:** 2022-08-30

**Authors:** Silvia Garcia-Cirera, Joan Calvet, Antoni Berenguer-Llergo, Edwards Pradenas, Silvia Marfil, Marta Massanella, Lourdes Mateu, Benjamin Trinité, Maria Llop, Marta Arévalo, Carlos Galisteo, Cristóbal Orellana, Rafael Gómez, María Nieves Gómez-Gerique, Inma Carmona, Bonaventura Clotet, Julià Blanco, Jordi Gratacós

**Affiliations:** 1 Rheumatology Department, Parc Taulí Hospital Universitari, Institut d’Investigació i Innovació Parc Taulí (I3PT), c/Parc Taulí S/N, Edifici VII Centenari Rheumatology Department, 08208 Sabadell, Spain; 2 Departament de Medicina, Universitat Autónoma de Barcelona (UAB), 08003 Barcelona, Spain; 3 Biostatistics and Bioinformatics Rheumatology Department, Institut d’Investigació i Innovació Parc Taulí (I3PT), 08208 Sabadell, Spain; 4 IrsiCaixa AIDS Research Institute, Germans Trias i Pujol Research Institute (IGTP), Can Ruti Campus, UAB, 08916 Badalona, Catalonia Spain; 5 University of Vic-Central University of Catalonia (UVic-UCC), 08500 Vic, Catalonia, Spain; 6 Laboratory Technician at Research Unit, Institut d’Investigació i Innovació Parc Taulí (I3PT), 08208 Sabadell, Spain

**Keywords:** Rheumatology, Infectious diseases, Rheumatic diseases

## Abstract

Limited data exists on SARS-CoV-2 sustained-response to vaccine in patients with rheumatic diseases. This study aims to evaluate neutralizing antibodies (nAB) induced by SARS-CoV-2 vaccine after 3 to 6 months from administration in Systemic Lupus Erythematosus (SLE) patients, as a surrogate of sustained-immunological response. This cross-sectional study compared nAB titre of 39 SLE patients and 37 Healthy individuals with no previous SARS-CoV-2 infection, who had all received a complete regimen of a mRNA SARS-CoV-2 vaccine within the last 3 to 6 months. We included four lines of SLE treatment including Not-treated, Hydroxychloroquine, immunosuppressive drugs and biological therapy. Glucocorticoids were allowed in all groups. Healthy and Not-treated individuals showed the highest levels of nAB. Treated patients presented lower nAB titres compared to Healthy: a 73% decrease for First-Line patients, 56% for Second-Line treatment and 72% for Third-Line. A multivariate analysis pointed to Glucocorticoids as the most associated factor with declining nAB levels (75% decrease) in treated SLE. Furthermore, a significant reduction in nAB titres was observed for Rituximab-users compared to Healthy subjects (89% decrease). Medium-term response of SLE patients to SARS-CoV-2 mRNA vaccines is negatively impacted in Glucocorticoids and Rituximab users. These findings might help to inform recommendations in vaccination protocols for SLE patients.

## Introduction

The spread of coronavirus disease 2019 (COVID-19) has resulted in a severe economic and health crisis worldwide. In this adverse scenario, vaccination programs have demonstrated to play a key role in fighting the global pandemic^1^. BNT162b2 (Pfizer-BioNTech) and mRNA-1273 (Moderna), both based on the novel Messenger RNA (mRNA) technology, were the first vaccines approved by the Food and Drug Administration and the European Medicines Agency^2,3^.

Patients with rheumatic diseases are considered a risk factor for community infections, although controversies exist regarding COVID-19 severity^4,5^. There still exists lack of data regarding vaccines' efficacy in vulnerable collectives with a compromised immune system, either due to a chronic pathology or to therapies targeting an autoimmune disease. Randomized controlled studies evaluating the efficacy of SARS-CoV-2 vaccines usually exclude patients of rheumatic autoimmune disease, so information about vaccines' efficacy in this group of subjects is limited^6^. There currently exists recommendations for anti-viral vaccinations of individuals under immunosuppressing therapies, including specific guidelines for SARS-CoV-2 vaccination provided by different scientific societies^7^.

A recently published systematic review evaluated SARS-CoV-2 infection and vaccination in rheumatic and musculoskeletal diseases. The study concluded no differences between specific diseases, a good general immunogenic response despite showing lower levels of antibodies compared to the general population and pointed to immunosuppressing therapies as the main source of impact on vaccines' response over disease condition, although this point remains controversial^5,8^. Their suggestions for future work included the separate study of specific pathologies in order confirm their findings. In this regard, Systemic Lupus Erythematosus (SLE) patients represent a highly interesting population, since they often display factors such as the deregulation of the type I interferon, the impairment of the lymphocyte functions and the use of immunosuppressive drugs. These factors might have an impact os the vaccines response, as previously described in the case of influenza A vaccination^5,9–11^. In addition, viral infections trigger both type I IFN production and B cell activation, two mechanisms frequently altered in SLE patients and, hence, likely to interfere in their immunological response to vaccines^10,12^.

Immune responses to SARS-CoV-2 vaccines involve humoral and cellular arms of adaptive immunity. While the specific contribution of each arm is not yet described, T-cell responses seem to be relevant for protection against severe disease, while antibodies seem to be directly related with the protection against SARS-CoV-2 infection^13^. Although different functions of antibodies could be responsible for their protective effect, neutralizing antibodies that bind to the spike (S) glycoprotein of SARS-CoV-2 and block viral entry into target cells appear to be the most relevant ones^14^. Several experimental and epidemiological studies on SARS-CoV-2 suggest that the titre of neutralizing antibodies (nAB) could be a useful as a surrogate for protection^15^, as they are for other viral infections.

The present study aims to evaluate the medium-term response to SARS-CoV-2 vaccination in SLE patients under different therapy regimes compared to healthy individuals. To do so, we quantified their levels of nAB from 3 to 6 months after their vaccination as a surrogate of protection against SARS-CoV-2 infection.

## Results

Neutralizing antibody titres were assessed for a total of 76 serum samples from 39 (51%) Lupus patients and 37 (49%) healthy Controls. Lupus patients were selected to equitably cover a wide range of treatment settings, that included Non-Treated patients and subjects under First-Line (Hydroxychloroquine, 10 patients, median daily dose 300 mg), Second-Line (10 patients: Methotrexate, n = 4 median weekly dose 15 mg; Azathioprine, n = 4, median daily dose 100 mg; and Mycophenolate Mofetil, n = 2) and Third-Line therapy (9 patients, Rituximab, n = 4 and Belimumab, n = 5) (Table 1 and Supplementary Table S1). Glucocorticoids users presented a median daily dose of 3 mg of methylprednisolone.

**Table 1:** Descriptive of subjects’ characteristics in the study according to disease and treatment status. Continuous variables are described by medians, minimum and maximum values; categorical variables are summarized using absolute frequencies and percentages. p-values are derived from a Mann–Whitney (continuous) or a Fisher's test (categorical variables).

		All n = 76	Control n = 37 (48.7%)	Not-treated n = 10 (13.2%)	First-Line n = 10 (13.2%)	Second-Line n = 10 (13.2%)	Third-Line n = 9 (11.8%)	p-value
Sex	Female	65 (85.5%)	30 (81.1%)	10 (100.0%)	10 (100.0%)	8 (80.0%)	7 (77.8%)	0.3315
	Male	11 (14.5%)	7 (18.9%)	0 (0.0%)	0 (0.0%)	2 (20.0%)	2 (22.2%)	
Age at sample extraction		46.0 (20.0, 82.8)	38.0 (20.0, 67.0)	56.8 (45.2, 82.8)	50.1 (34.6, 75.1)	51.5 (45.0, 81.8)	45.4 (34.8, 70.0)	< 0.0001
Time from vaccination to sample extraction (months)		3.4 (2.3, 5.5)	3.3 (2.6, 4.3)	3.3 (2.3, 4.3)	3.4 (3.3, 4.1)	3.9 (3.3, 4.4)	3.5 (3.1, 5.5)	0.0064
Vaccine type	Moderna	44 (57.9%)	17 (45.9%)	5 (50.0%)	7 (70.0%)	6 (60.0%)	9 (100.0%)	0.0329
	Pfizer	32 (42.1%)	20 (54.1%)	5 (50.0%)	3 (30.0%)	4 (40.0%)	0 (0.0%)	
Time of disease evolution (years)		14.3 (2.3, 39.4)		17.8 (7.2, 36.3)	11.7 (6.3, 36.3)	13.8 (2.3, 36.3)	16.2 (4.3, 39.4)	0.5892
Any Concomitant Treatment	No	20 (51.3%)		9 (90.0%)	7 (70.0%)	3 (30.0%)	1 (11.1%)	0.0016
	Yes	19 (48.7%)		1 (10.0%)	3 (30.0%)	7 (70.0%)	8 (88.9%)	
Concomitant Treatment with Clucocorticoids	No	30 (76.9%)		9 (90.0%)	7 (70.0%)	7 (70.0%)	7 (77.8%)	0.7575
	Yes	9 (23.1%)		1 (10.0%)	3 (30.0%)	3 (30.0%)	2 (22.2%)	
SLEDAI 2 years average	SLEDAI < 4	36 (92.3%)		9 (90.0%)	10 (100.0%)	9 (90.0%)	8 (88.9%)	
	SLEDAI [4, 6)	3 (7.7%)		1 (10.0%)	0 (0.0%)	1(10.0%)	1 (11.1%)	
SLEDAI nearest to vaccination	SLEDAI < 4	34 (87.2%)		8 (80.0%)	9 (90.0%)	9 (90.0%)	8 (88.9%)	
	SLEDAI [4, 6)	3 (7.7%)		1 (10.0%)	0 (0.0%)	1 (10.0%)	1 (11.1%)	
	SLEDAI 6 +	2 (5.1%)		1 (10.0%)	1 (10.0%)	0 (0.0%)	0 (0.0%)	

The median age was 46 years old, although Controls were younger than Lupus patients (38 vs 53 years old median, respectively). Among Lupus patients, those treated with Third-Line therapy showed a shorter age (45 years old median) while Not-Treated patients were the oldest subjects of the series (57 years old median). Females were majority in this study (86%) with a similar proportion in Controls and patients (81 and 90%, respectively). Moderna and Pfizer vaccines had been administered roughly equitably in the Control group (46% and 54%, respectively), while Lupus patients had been prevalently inoculated with Moderna's (69%). Subject groups were homogenous regarding time interval between vaccination and sample extraction (3.4 months median; Table 1).

Regarding SLE clinical parameters, only two patients (5%) showed an SLEDAI score above 6 in their nearest measurement to the extraction date, and none of them exceed this value as 2-years SLEDAI average. Disease evolution time was 14 years’ median, ranging from 12 in First-Line to 18 years in Not-Treated patients. Patient groups were heterogeneous regarding their current regimes of concomitant therapy (from 10% in non-Treated to 89% in Third-Line treated patients), which near half of the times included therapy with Glucocorticoids (23%; Table 1).

**Figure 1:**
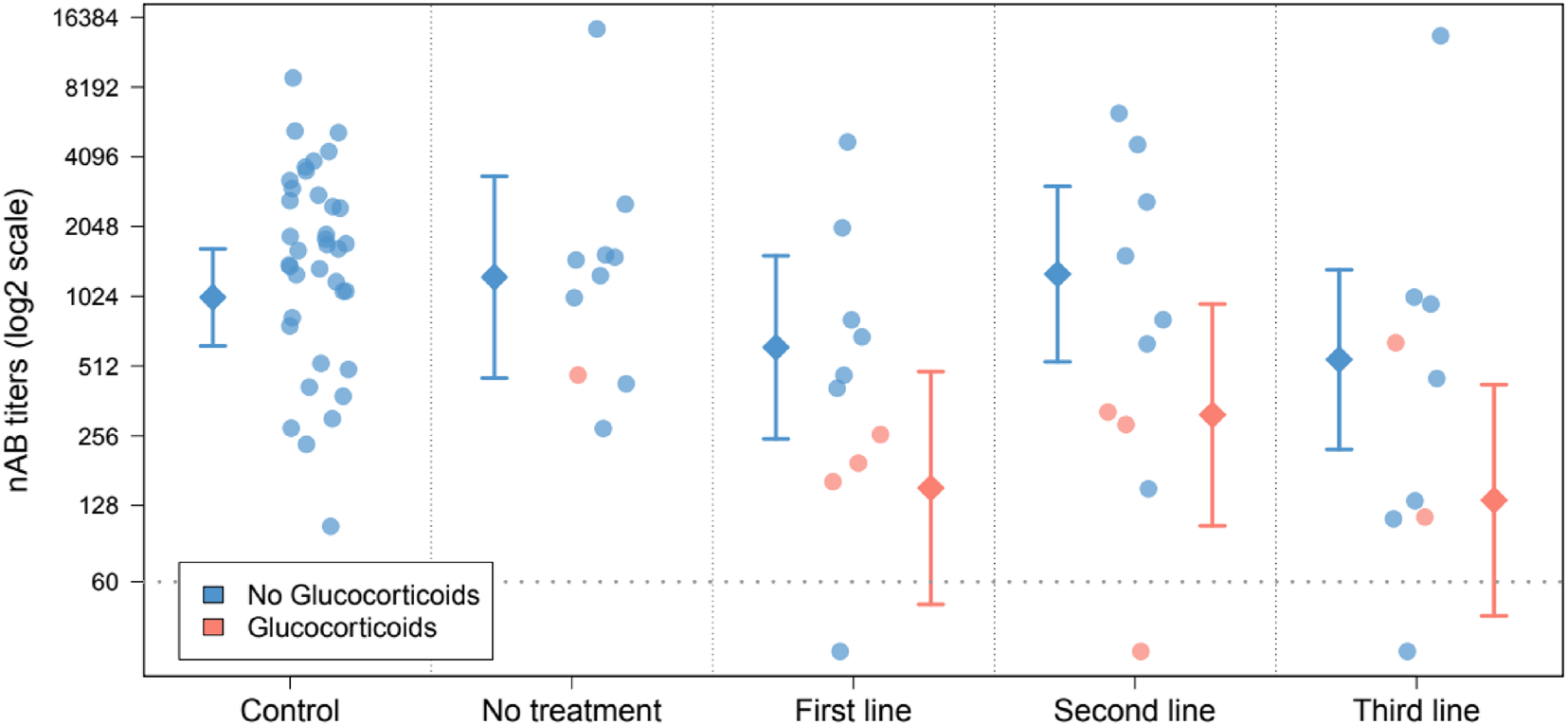
Neutralizing antibody (nAB) titre after SARS-CoV-2 vaccination in Healthy individuals (Controls) and Systemic Lupus Erythematosus (SLE) patients under different therapy regimens. Diamond-shaped symbols and their associated segments represent adjusted means and 95% confidence intervals of antibody titres. Estimations are derived from a linear model in which sex, age, time from vaccination, vaccine type and corticoids therapy were included as covariates for statistical control. Antibody titres were log2-transformed in order to fit the assumptions of the model and are represented in log2-scale. The horizontal dotted line indicates the detection threshold for the determinations (60). The estimation of the decrease in nAB titres in Lupus patients associated to Glucocorticoids therapy is 75% (Fold-Change = 0.25, 95% Confidence Interval = 0.10–0.63, p = 0.0037).

The highest nAB titres corresponded to Healthy Control subjects (1638.0 titre median), which were similar to SLE patients not currently under treatment (1361.5 titre median). Treated patients showed considerably lower levels of nAB levels compared to Controls, a decrease estimated in 73% in patients receiving Hydroxychloroquine (First-Line therapy; p = 0.0135), 56% for patients in Second-Line (p = 0.2218) and 72% for Third-Line treated patients (p = 0.0104) (Fig. 1, Table 2 and Supplementary Table S2). Three Lupus patients (10%) showed titres of nAB below the detection threshold, while the response to vaccine was measurable for all non-Treated patients and Healthy Controls (Fig. 1).

**Table 2:** Univariate association of neutralizing antibody (nAB) levels after SARS-CoV-2 vaccination with Disease/Treatment status and demographic and clinical parameters. Association with continuous variables are estimated using Spearman Correlation Coefficients (SCC). Categorical variables, antibody levels are summarized using group medians. Range between brackets correspond to 95% confidence intervals computed with 1.000 boostrap resamples. p-values are derived from the associated SCC for continuous variables, while a Mann–Whitney (binary) or a Kruskal test (Disease/Treatment group) were used for categorical variables. SCC: Spearman Correlation Coefficient; 95% CI: 95% confidence interval.

		Median/SCC [95% CI]	p-value
Disease-treatment group	Control	1638.0 [1182.0, 2459.0]	0.0220
	Not-treated	1361.5 [429.0, 2555.0]	0.0220
	First-Line	438.5 [162.0, 2022.0]	0.0220
	Second-Line	724.0 [151.0, 4620.0]	0.0220
	Third-Line	452.0 [112.0, 1016.0]	0.0220
Sex	Female	1256.0 [809.0, 1612.0]	0.5304
	Male	762.0 [151.0, 3559.0]	0.5304
Age at sample extraction		−0.284 [−0.500, −0.058]	0.0128
Time from vaccination (months)		−0.375 [−0.575, −0.159]	0.0008
Vaccine type	Moderna	1136.0 [645.0, 1612.0]	0.6662
	Pfizer	1129.5 [467.0, 1856.0]	0.6662
Time of Disease Evolution (years)		−0.174 [−0.480, 0.133]	0.2882
Any Concomitant Treatment	No	909.5 [429.0, 1546.0]	0.1774
	Yes	452.0 [162.0, 1016.0]	0.1774
Concomitant Treatment with Glucocorticoids	No	978.5 [467.0, 1529.0]	0.0057
	Yes	259.0 [114.0, 468.0]	0.0057

Regarding other factors potentially associated with the vaccine response, nAB titres were inversely correlated with subject's age (Spearman Correlation = −0.284, p = 0.0128) and time from vaccination (Spearman Correlation = −0.375, p = 0.0008). A 74% decrease was also observed in patients treated with Glucocorticoids compared to non-users (p = 0.0057). No significant association was found between nAB titres and sex nor vaccine type (Fig. 1 and Table 2).

Figure 1 and Table 3 show the results from comparing nAB titres across subjects’ groups after statistical control for potential confounders: sex, age, time from vaccination, vaccine type and Glucocorticoids users (see complete model in Supplementary Table S3). This analysis revealed that, although differences between Control and First- and Third-Line patients were retained with a similar magnitude effect to those observed in the univariate setting (First-Line: 70% decrease, p = 0.0179; Third-Line: 73% decrease, p = 0.0119), most of these differences were explained by the strong effect that Glucocorticoids therapy had on the nAB titres, which accounted for a 75% decrease compared to patients not prescribed with Glucocorticoids (p = 0.0037; Fig. 1, Supplementary Figure S1, Supplementary Table S3). As a result, declines in nAB titres were of a substantially higher magnitude in patients treated with Glucocorticoids than those observed in non-users (First-Line: 85%, p = 0.0022; Third-Line: 87%, p = 0.0015), where differences did not reach statistical significance (First-Line: 39%, p = 0.3136; Third-Line: 46%, p = 0.2246). Second-Line patients using Glucocorticoids also showed lower nAB levels compared to Healthy individuals although, in this case, the decrease was smaller and not statistically significant (69%, p = 0.0519) (Fig. 1, Table 3 and Supplementary Table S3; see Supplementary Table S4 for the rest of pairwise comparisons). Regarding the rest of covariates, the negative correlation observed in the univariate analysis for time from vaccination was retained after adjustment by confounders, which was estimated as a 48% decrease per month (Partial Correlation = −0.267, p = 0.0277). No statistically significant association was found for sex, age nor vaccine type (Supplementary Table S3).

**Table 3:** Neutralizing antibody (nAB) levels of Systemic Lupus Erythematosus (SLE) patients after SARS-CoV-2 vaccination compared to Healthy subjects. Table cells show the Fold-Changes (FC), 95% Confidence Intervals (95% CI) and p-values derived from comparing antibody titers between each treatment line group and the Healthy subjects, which are taken as reference. Comparisons are performed for subjects receiving and not currently receiving Glucocorticoids’ therapy (Corticoids and No-Corticoids columns), as well as for all patients overall (All columns). Estimations and p-values are derived from a linear model in which sex, age, time from vaccination, vaccine type and corticoids therapy were included as covariates for statistical control. FC: Fold-change; 95% CI: 95% confidence interval.

	All		No-Glucocorticoids		Glucocorticoids	
	FC [95% CI]	p-value	FC [95% CI]	p-value	FC [95% CI]	p-value
Control (Reference)	1	–	1	–	1	–
Not-treated	0.61 [0.19, 1.94]	0.3950	1.22 [0.42, 3.52]	0.7059	0.30 [0.07, 1.25]	0.0972
First-Line	0.30 [0.11, 0.81]	0.0179	0.61 [0.23, 1.62]	0.3136	0.15 [0.05, 0.49]	0.0022
Second-Line	0.63 [0.23, 1.68]	0.3463	1.26 [0.47, 3.40]	0.6437	0.31 [0.10, 1.01]	0.0519
Third-Line	0.27 [0.10, 0.74]	0.0119	0.54 [0.20, 1.48]	0.2246	0.13 [0.04, 0.45]	0.0015

A detailed analysis of specific therapy agents suggested a differential impact of drugs prescribed within Second- and Third-Lines. In the Third-Line group and in the absence of Glucocorticoids, an 89% decrease in antibody titres was observed in patients treated with Rituximab compared to Healthy Controls (p = 0.0008), while only a modest 7% was displayed by those received Belimumab (p = 0.8982). Patients under Second-Line therapy also showed different impacts regarding drug prescription without Glucocorticoids; these differences were estimated as a 39% decrease for Azathioprine and 8% for Methotrexate, although none of them reached statistical significance (p-values 0.4556 and 0.8935, respectively). Differences displayed by patients under Azathioprine were magnified by the effect of Glucocorticoids treatment (79% decrease, p = 0.0282). Although suggestive, these results at the drug level are exploratory in nature and should be interpreted with caution due to the low sample size available for the analyses (from 2 to 4 patients in the treated patients’ group) (Supplementary Figure S2 and Supplementary Table S5).

## Discussion

Our evaluation of the response to mRNA SARS-CoV-2 vaccination showed that, in the medium-term (3 to 6 months), the levels of nABs in patients under treatment for SLE is of a lower magnitude compared to those in controls or in non-treated patients. These lower titres in nAB are predominantly explained by the use of Glucocorticoids and Rituximab and, possibly and to a lesser extent, by the use of other immunosuppressive drugs for controlling the severity of the disease. This decrease in nAB titres are likely to be correlated with the probability of infection and, hence, might have implications in the vaccination protocols establish for SLE patients.

A higher prevalence of non-response to SARS-CoV-2 vaccination has been reported in individuals with rheumatic diseases treated with Glucocorticoids, Mycophenolate-Mofetil or Rituximab^16^. Furthermore, a hampered antibody responses to SARS-CoV-2 vaccines have been found in patients of autoimmune diseases treated with Methotrexate, Mycophenolate-Mofetil or Rituximab, and the authors suggested an extended treatment modification to improve the vaccine-induced immunogenicity^17^. Many previous studies have identified a lower short-term response to SARS-CoV-2 vaccines as measured by nAB levels in subjects with autoimmune conditions compared to healthy controls, as well as a decrease percentage of responders^16^. The immunosuppressive treatments prescription in rheumatic disease patients have also been associated to lesser levels of nAB shortly after SARS-CoV-2 vaccination, as well as to therapy with Glucocorticoids and directly to SLE condition^18,19^. Overall, these previous studies agree in reporting a weaker response to vaccination in patients with rheumatic diseases, which might be more associated to treatments with immunosuppressive drugs rather than to the specific conditions of the disease itself^20^, although SLE might negatively impact to the immunogenic response^19^. Among them, Rituximab has been the more extensively studied and it has been linked to lower humoral antibody and T cell responses shortly (no more than two months) after vaccination^21,22^. Therefore, the impact of rheumatic inflammatory diseases on SARS-CoV-2 vaccine-induced response was previously evaluated at a short-term period. In our data, though, no differences in the vaccine response were found between healthy individuals and not-treated SLE patients, and this observation points to the different drug regimens and, specially, the use of Glucocorticoids or Rituximab, as the predominant cause of a lower response to the vaccine in SLE patients. Of note, therapy regimens were considered as a surrogate of SLE severity in our study by design and, therefore, it was not possible to discriminate in our data the impact of the different therapeutic options from that of the SLE conditions with a higher severity (i.e., First-, Second- and Third-Line groups).

Specifically focused on SLE studies, approximately 30% of patient experienced a low response to SARS-Cov-2 vaccines^23^. Furthermore, a quantifications of antibodies against SARS-CoV-2 in patients with Rheumatoid Arthritis (RA) and SLE after two doses of BNT162b2 resulted, in a previous work, in a 23% of patients with no detectable serological response to the vaccine. A poor humoral immune response has also been described for SLE patients treated with Glucocorticoids and immunosuppressive drugs after a single dose of mRNA vaccines^24^. SLE patients under Methotrexate or Mycophenolate-Mofetil regimes have also showed a lower antibody response against SARS-CoV-2 fifteen days after the second dose^25^, although current evidence about the effect of Methotrexate on humoral response is not consistent^26,27^. None of the works mentioned above evaluated nAB as a measure of response to the vaccine. Another study on SLE patients, though, confirmed the use of Prednisone and Mycophenolate-Mofetil as independently associated with the absence of nABs six weeks after a second dose of the Sinovac-Coronovac vaccine^28^. This study suggested a positive effect of Hydroxychloroquine on the early immunogenic response, although this observation was not supported in previous works nor in our data^18^.

Previous studies on SLE patients are in partial agreement with the findings reported in the present work, where Glucocorticoids and Rituximab seemed to interfere in the achievement and maintenance of an optimal nAB response to SARS-CoV-2 vaccination. As it is reported in previous studies, peripheral B cell depletion after Rituximab courses impairs the immunogenic response to SARS-CoV-2 vaccine^29^. This data supported our results where Rituximab exerted a negative impact in medium-term vaccine immunogenicity. We observed a decrease in nAB titres in SLE patients under Glucocorticoids’ regimen as a concomitant therapy. It is important to highlight that, despite the median daily dose was 3 mg of Methylprednisolone, the medium-term nAB titres were significantly lower compared to non-Glucocorticoid users. Previous studies and guidelines described high doses of Glucocorticoids as a risk factor for reduced vaccine response^30^; our observation suggest that Glucocorticoid at a lower doses may also decrease this acquired immunogenic response in the medium-term^18^. Only two patients under Mycophenolate-Mofetil therapy were evaluated in our study, although it is worthy to note that one of them did not present detectable nAB titres. We did not observe Methotrexate as an obstacle for obtaining an optimal medium-term response to mRNA vaccines. Discrepancies exists in previous studies evaluating the effect of this drug in the short-term response to vaccines^26,27^. In our series, no subject under Methotrexate had received Glucocorticoids and we did not detect a decrease in its nAB titre. We did not observe either an impact of Azathioprine or Hydroxychloroquine in medium-term immunogenic response when administered alone without concomitant Glucocorticoids. To interpret the results provided, it is important to consider that our study evaluates the preservation of the immunological response in the medium-term, that is, within a period between 3 and 6 months after the second vaccination dose. In contrast, all previously published studies assessed the short-term response to vaccination (4 to 6 weeks after completion of the schedule), which might explain some of the results differences observed between these works and our own. Although it was not possible to stablish cut off points for nAB due to technical concerns, it is accepted that higher levels of nAB provides a more protection against SARS-CoV-2 infection^31^. In this regard, the use of nAB titres as a surrogate of sustained immunogenic response might help to define specific vaccination schedules for SLE patients, especially for those under Glucocorticoids and Rituximab regimens.

### Strengths and limitations

The main limitation of our study arises from its cross-sectional nature, which limits the ability to establish causality. Also, SLE patients were recruited at a single clinical centre, which limited the sample size available for analyses. This is especially true for the results at the level of specific treatments within therapy lines (2 to 4 samples in the treatment groups) where results, although appealing, should be interpreted with caution. These limitations determine the exploratory nature of our findings until they are confirmed in larger and independents series of patients. Also, and although symptomatic SARS-CoV-2 infection was discarded for all participants by interview and review of clinical history, there was not availability of serology studies or history of anti-N protein antibodies for these subjects. Hence, we cannot discard the inadvertent inclusion of individuals with previous asymptomatic COVID-19 infection, a weakness that is shared by many previous studies.

On the other hand, SLE patients were selected to represent a wide range of treatment regimes, which allowed to evaluate nAB titres in different clinical scenarios. In our design, patients' therapies were considered as a surrogate of SLE severity and, although no differences in the vaccine response were found between healthy subjects and Not-treated SLE patients (a group that includes the patients with the less severe presentation), our study did not provide data to differentiate the effects of SLE conditions with higher severity from their prescribed immunosuppressive therapy.

There also exist technical concerns regarding the measurements of nAB in patients’ plasma. Although nAB quantification were conducted with a set of tools and protocols previously validated^32^, the procedure was not standardized to International Units per millilitre (IU/mL), as recommended by World Health Organization (WHO). These methods may be different to those used in other laboratories and, therefore it is not possible to calibrate and harmonize our results for comparison purposes, or to establish meaningful cut offs defining different levels of vaccination response that can be extrapolated to other studies.

As described above, previous work exists describing the impact of treatment in patients of autoimmune disease on SARS-CoV-2 vaccination. Most of these studies, though, jointly analysed different pathologies, focused on the short-term response (2 to 6 weeks) after inoculation or did not evaluate nAB specifically as a surrogate of risk infection. Our work is, to our knowledge, the first studying the preservation in the medium-term (3 to 6 months) of the response induced by mRNA SARS-CoV-2 vaccines, using abundance of plasma nABs as measure of this response, and focused specifically in SLE patients treated with a variety of therapy regimens.

## Conclusion

Medium-term response of SLE patients to SARS-CoV-2 vaccination, as measured by the titre of nABs, may be compromised by Glucocorticoids use and other prescribed treatments aimed to control the severity of the disease. This reduced response is likely translates into a higher probability of COVID-19 infection. The confirmation of these findings in larger longitudinal series of SLE patients, might help to inform recommendations in vaccination protocols for SLE patients, especially for those under Glucocorticoids and/or Rituximab regimens.

## Methods

### Study design and subjects

The present work is a cross-sectional study that compares the nAB levels in the serum of 39 Systemic Lupus Erythematosus (SLE) patients and 37 healthy individuals (Controls) after vaccination against the SARS-CoV-2. None of the subjects had been previously exposed to the SARS-CoV-2, and all had received a complete two-doses schedule of BNT162b2 (Pfizer) or mRNA-1237 (Moderna) vaccines within the 3 to 6 months prior to sample extraction.

SLE patients included: 10 patients with no requirement of active SLE-specific therapy, possibly under concomitant treatment of Glucocorticoids (Not-Treated); 10 patients with mild to moderate SLE with indication of treatment with Hydroxychloroquine (First-Line); 10 patients with moderate to severe SLE with prescription of immunosuppressive drugs as Methotrexate, Azathioprine, or Mycophenolate Mofetil (Second-Line); and, finally, 9 SLE patients under biological treatment (Third-Line) with Belimumab or Rituximab. A third line treatment patient had been previously excluded from the study as it had received a complete vaccination schedule before its sample extraction (Table 1 and Supplementary Table S1). Glucocorticoids were permitted in all groups as a concomitant treatment.

After a subject was selected as potential participant, a member of the research team contacted them telephonically to expose the study and to offer their enrolment. At the inclusion data, the study was again described to the subject, who signed the informed consent and provided the blood sample. The laboratory technicians processed and conserved the sample at −80ºC until the serologic quantifications. We selected a control group of subjects from the KING study (Hospital Germans Trias i Pujol, Badalona Spain, HUGTiP, PI-20-122 and PI-20-217), without comorbidities and no previous virus exposure, who had also received a complete regimen of BNT162b2 (Pfizer) or mRNA-1237 (Moderna) vaccination within the last 3 to 6 months. This study was approved by the Local Ethical Committee at the Hospital Universitari Parc Taulí, Sabadell (2021/5093). All patients included were verbally informed and signed informed consent and all methods were performed in accordance with the relevant guidelines and regulations.

### Assessments

We collected the following subjects’ information: age, sex, type of vaccine (BNT162b2 (Pfizer) or mRNA-1237 (Moderna)), date of vaccination (second dose) and date of sample extraction. For SLE patients’ additional clinical parameters were collected: disease evolution time, last SLE Disease Activity Index (SLEDAI) measured before sample extraction, the average of the SLEDAI measurements obtained over the last two years, and any concomitant treatment prescribed in addition to their regimen.

### Neutralization assays

HEK293T cells (Integral Molecular (Cat# C-HA101), presumably of female origin) overexpressing wild-type human ACE-2 (Integral Molecular, USA) were used as target for SARS-CoV-2 spike expressing pseudovirus infection. Cells were maintained in T75 flasks with Dulbecco′s Modified Eagle′s Medium (DMEM) supplemented with 10% foetal bovine serum and 1 μg/mL puromycin).

HIV-based pseudoviruses expressing the SARS-CoV-2 S protein and luciferase were constructed using the defective HIV plasmid, pNL4-3.Luc.R-.E-. The plasmid was obtained from the NIH AIDS Reagent Program. Expi293F cells were transfected using ExpiFectamine293 Reagent (Thermo Fisher Scientific, Waltham, MA, USA) with pNL4-3. Luc.R-.E- and SARS-CoV-2.SctΔ19 (Wuhan); at an 8:1 ratio, respectively^33^. Control pseudoviruses were generated by replacing the S protein expression plasmid with a vesicular stomatitis virus (VSV)-G protein expression plasmid as previously reported^34^. Supernatants were harvested 48 h after transfection, filtered at 0.45 μm, frozen, and titrated on HEK293T cells overexpressing wild-type human ACE-2.

Neutralization assays were performed in duplicate. Briefly, 200X the median tissue culture infectious dose (TCID50) of pseudovirus was preincubated with three-fold serial dilutions (1/60–1/14,580) of heat-inactivated plasma samples in Nunc 96-well cell culture plates (Thermo Fisher Scientific) for 1 h at 37 °C. Then 2 × 104 HEK293T/hACE2 cells treated with DEAE-Dextran (Sigma-Aldrich, St. Louis, MO, USA) were added. Results were read after 48 h using an EnSight Multimode Plate Reader and BriteLite Plus Luciferase reagent (Perkin Elmer, Waltham, MA, USA). The results were normalized and the ID50 (the reciprocal dilution inhibiting 50% of the infection) was calculated by plotting the log of plasma dilution versus response and fitting to a 4-parameter equation in Prism 8.4.3 (GraphPad Software, San Diego, CA, USA). This neutralization assay has been previously validated in a large subset of samples^32^.

### Statistical methods

For descriptive purposes, we used absolute and relative frequencies for categorical variables while medians, minimum and maximum values and interquartile ranges were used for continuous measurements. Univariate differences in nAB between subject groups were assessed by non-parametric techniques that included Mann–Whitney tests (binary variables), Kruskal test (categorical variables with more than one category) and Spearman Correlation (continuous variables). In these analyses, 95% confidence intervals (95% CI) for Fold-Changes between groups were computed using 1.000 bootstrap resamples stratified by condition group. Multivariate associations were assessed using linear models where sex, age at sample extraction, time from vaccination, vaccination type and glucocorticoids therapy were considered as covariates for statistical control. For doing so, antibody quantifications were log2-transformed in order to fulfil the assumptions of the model. Quantifications that did not reached the minimum detection threshold (threshold = 60) were previously assigned to a value equal to half this threshold (antibody titre = 30). Partial Correlations adjusted group means at the original scale (after undoing the log2 transformation) and FCs and their 95% CI were retrieved from the models to express the magnitude of the effects. Statistical significance was assessed using Wald tests derived from the models. Results were represented graphically using a stripchart in which the group means, and 95% CI were also included after adjustment by confounders. Statistical significance was set at the 5% threshold. All analyses were conducted using R.

## Supplementary Information

Supplementary Information 1.

Supplementary Information 2.

## Data Availability

The data underlying this article is shared as Supplementary File.
