# Supplementary material for: Glucocorticoids’ treatment impairs the medium-term immunogenic response to SARS-CoV-2 mRNA vaccines in Systemic Lupus Erythematosus patients
Source: Sci Rep. 2022 Aug 30;12:14772. doi: 10.1038/s41598-022-18996-x (PMC9427088; doi:10.1038/s41598-022-18996-x)
Supplement: Supplementary file 1 — Supplementary Information 1. [file 41598_2022_18996_MOESM1_ESM.doc]

**Table S1.** Descriptive of subjects characteristics in the study according to disease and treatment status and Glucocorticoids therapy. Cells show absolute frequencies and by-row percentages.

|  | | | **No Glucocorticoids** | **Glucocorticoids** | **All** |
| --- | --- | --- | --- | --- | --- |
| **Healthy** | **Control** | | 37 (100.0%) | 0 (0.0%) | 37 (100%) |
| **SLE** | **Not-Treated** | | 9 (90.0%) | 1 (10.0%) | 10 (100%) |
|  | **First-Line** | **Hydroxychloroquine** | 7 (70.0%) | 3 (30.0%) | 10 (100%) |
|  | **Second-Line** | **Methotrexate** | 4 (100.0%) | 0 (0.0%) | 4 (100%) |
|  |  | **Azathioprine** | 2 (50.0%) | 2 (50.0%) | 4 (100%) |
|  |  | **Mycophenolate Mofetil** | 1 (50.0%) | 1 (50.0%) | 2 (100%) |
|  | **Third-Line** | **Belimumab** | 4 (80.0%) | 1 (20.0%) | 5 (100%) |
|  |  | **Rituximab** | 3 (75.0%) | 1 (25.0%) | 4 (100%) |

**SLE:** Systemic Lupus Erythematosus

**Table S2.** Univariate pairwise comparisons of neutralizing antibody (nAB) levels of healthy Controls and Systemic Lupus Erythematosus (SLE) patients after SARS-CoV-2 vaccination. Cells below the table's diagonal show the Fold-Changes and corresponding 95% Confidence Intervals (between brackets), which are estimated as the ratio of medians between antibody titers between each subject group (rows) and every other group defined in the study (columns, which are taken as reference). Cells above the diagonal show the p-values associated with the corresponding comparison. For confidence intervals, a bootstrap procedure was conducted with 1.000 resamples stratified by comparison group. Statistical significance was derived from pairwise Mann-Whitney tests.

|  | **Reference** | | | | |
| --- | --- | --- | --- | --- | --- |
|  | **Control** | **Not-Treated** | **First-Line** | **Second-Line** | **Third-Line** |
| **Control** |  | 0.38 | 0.01 | 0.22 | 0.01 |
| **Not-Treated** | 0.83 [0.27, 1.29] |  | 0.07 | 0.55 | 0.05 |
| **First-Line** | 0.27 [0.12, 0.82] | 0.32 [0.13, 1.12] |  | 0.47 | 0.71 |
| **Second-Line** | 0.44 [0.17, 1.90] | 0.53 [0.19, 2.73] | 1.65 [0.40, 9.11] |  | 0.39 |
| **Third-Line** | 0.28 [0.06, 0.70] | 0.33 [0.07, 1.01] | 1.03 [0.14, 3.66] | 0.62 [0.06, 2.41] |  |

**Table S3.** Complete model for assessment of neutralizing antibody (nAB) levels after SARS-CoV-2 vaccination across Disease/Treatment subject groups. Results are derived from a linear model in which sex, age, time from vaccination, vaccine type and Glucocorticoids therapy were included as covariates for statistical control. Antibody titers was log2-transformed in order ot fit the assumptions of the model. Statistical significance was assessed using Wald tests (for regression coefficients) and F-tests (for overall significance of covariates). Partial Correlation for Time from Vaccination was estimated as -0.267 (95% Confidence Interval from -0.475 to -0.031).

|  | | **Beta [95%CI]** | **FC [95%CI]** | **Wald test p-values** | **F-test p-value** |
| --- | --- | --- | --- | --- | --- |
| **Disease-Treatment Group** | **Control** | Ref. | | | 0.4059 |
|  | **Not-Treated** | 0.289 [-1.235, 1.814] | 1.22 [0.42, 3.52] | 0.7059 |  |
|  | **First-Line** | -0.717 [-2.126, 0.692] | 0.61 [0.23, 1.62] | 0.3136 |  |
|  | **Second-Line** | 0.333 [-1.099, 1.766] | 1.26 [0.47, 3.40] | 0.6437 |  |
|  | **Third-Line** | -0.892 [-2.344, 0.561] | 0.54 [0.20, 1.48] | 0.2246 |  |
| **Sex** | **Female** | Ref. | | |  |
|  | **Male** | -0.627 [-1.871, 0.617] | 0.65 [0.27, 1.53] | 0.3181 |  |
| **Age at Sample Extraction** | | -0.017 [-0.052, 0.019] | 0.99 [0.96, 1.01] | 0.3489 |  |
| **Time from Vaccination (months)** | | -0.948 [-1.788, -0.107] | 0.52 [0.29, 0.93] | 0.0277 |  |
| **Vaccine Type** | **Moderna** | Ref. | | |  |
|  | **Pfizer** | -0.076 [-0.933, 0.781] | 0.95 [0.52, 1.72] | 0.8599 |  |
| **Concomitant Treatment with Corticoids** | **No** | Ref. | | |  |
|  | **Yes** | -2.018 [-3.357, -0.678] | 0.25 [0.10, 0.63] | 0.00373 |  |

**Beta:** regression coefficients; **FC:** Fold-Change; **95%CI:** 95% confidence interval; **Ref.:** Level reference for estimation of regression coefficients and Fold-Changes.

**Table S4.** Pairwise comparisons of neutralizing antibody (nAB) levels of Systemic Lupus Erythematosus (SLE) patients after SARS-CoV-2 vaccination. Cells below the table's diagonal show the Fold-Changes (FC) and 95% Confidence Intervals (between brackets), which are estimated as the ratio between antibody titers between each subject group (rows) and every other group defined in the study (columns, which are taken as reference). Cells above the diagonal show the p-values associated with the corresponding comparison. Results are derived from a linear model in which sex, age, time from vaccination, vaccine type and Glucocorticoids therapy were included as covariates for statistical control. Antibody titers were log2-transformed in order ot fit the assumptions of the model. Statistical significance was assessed using Wald tests.

|  | **Reference** | | | |
| --- | --- | --- | --- | --- |
|  | **Not-Treated** | **First-Line** | **Second-Line** | **Third-Line** |
| **Not-Treated** |  | 0.22 | 0.96 | 0.2 |
| **First-Line** | 0.50 [0.16, 1.53] |  | 0.18 | 0.83 |
| **Second-Line** | 1.03 [0.32, 3.32] | 2.07 [0.71, 6.03] |  | 0.13 |
| **Third-Line** | 0.44 [0.13, 1.54] | 0.89 [0.29, 2.72] | 0.43 [0.14, 1.30] |  |

**Table S5.** Neutralizing antibody (nAB) levels of Systemic Lupus Erythematosus (SLE) patients after SARS-CoV-2 vaccination compared to Healthy subjects. Table cells show the Fold-Changes (FC), 95% Confidence Intervals (95%CI) and p-values derived from comparing antibody titers between each treatment group and Healthy subjects, which are taken as reference. Comparisons are performed for subjects receiving and not currently receiving Glucocorticoids therapy (*Glucocorticoids* and *No-Glucocorticoids* columns), as well as for all patients overall (*All* columns). Estimations and p-values are derived from a linear model including treatment group and Glucocorticoids therapy as explanatory variables. Empty cells denote comparisons not evaluated due to only one sample or no samples available in the corresponding group.

|  | | | **All** | | **No-Glucocorticoids** | | **Glucocorticoids** | |
| --- | --- | --- | --- | --- | --- | --- | --- | --- |
|  |  |  | **FC [95%CI]** | **P-value** | **FC [95%CI]** | **P-value** | **FC [95%CI]** | **P-value** |
| **Healthy Controls (Reference)** | | | 1.00 | - | 1.00 | - | 1.00 | - |
| **SLE** | **No treatment** | | 0.55 [0.22, 1.37] | 0.1932 | 1.00 [0.43, 2.31] | 0.9991 |  |  |
|  | **First-Line Treatment** | **Hydroxychloroquine** | 0.24 [0.10, 0.57] | 0.0016 | 0.45 [0.19, 1.08] | 0.0717 | 0.13 [0.05, 0.39] | 0.0003 |
|  | **Second-Line Treatment** | **Methotrexate** | 0.50 [0.13, 1.88] | 0.3027 | 0.92 [0.27, 3.15] | 0.8935 |  |  |
|  |  | **Azathioprine** | 0.33 [0.10, 1.14] | 0.0790 | 0.61 [0.16, 2.28] | 0.4556 | 0.18 [0.05, 0.68] | 0.0122 |
|  |  | **Mycophenolate Mofetil** |  |  |  |  |  |  |
|  | **Third-Line Treatment** | **Belimumab** | 0.51 [0.16, 1.61] | 0.2446 | 0.93 [0.30, 2.88] | 0.8982 |  |  |
|  |  | **Rituximab** | 0.06 [0.02, 0.21] | < 0.0001 | 0.11 [0.03, 0.39] | 0.0008 |  |  |

**SLE:** Systemic Lupus Erythematosus; **FC:** Fold-change; **95%CI:** 95% Confidence intervals.


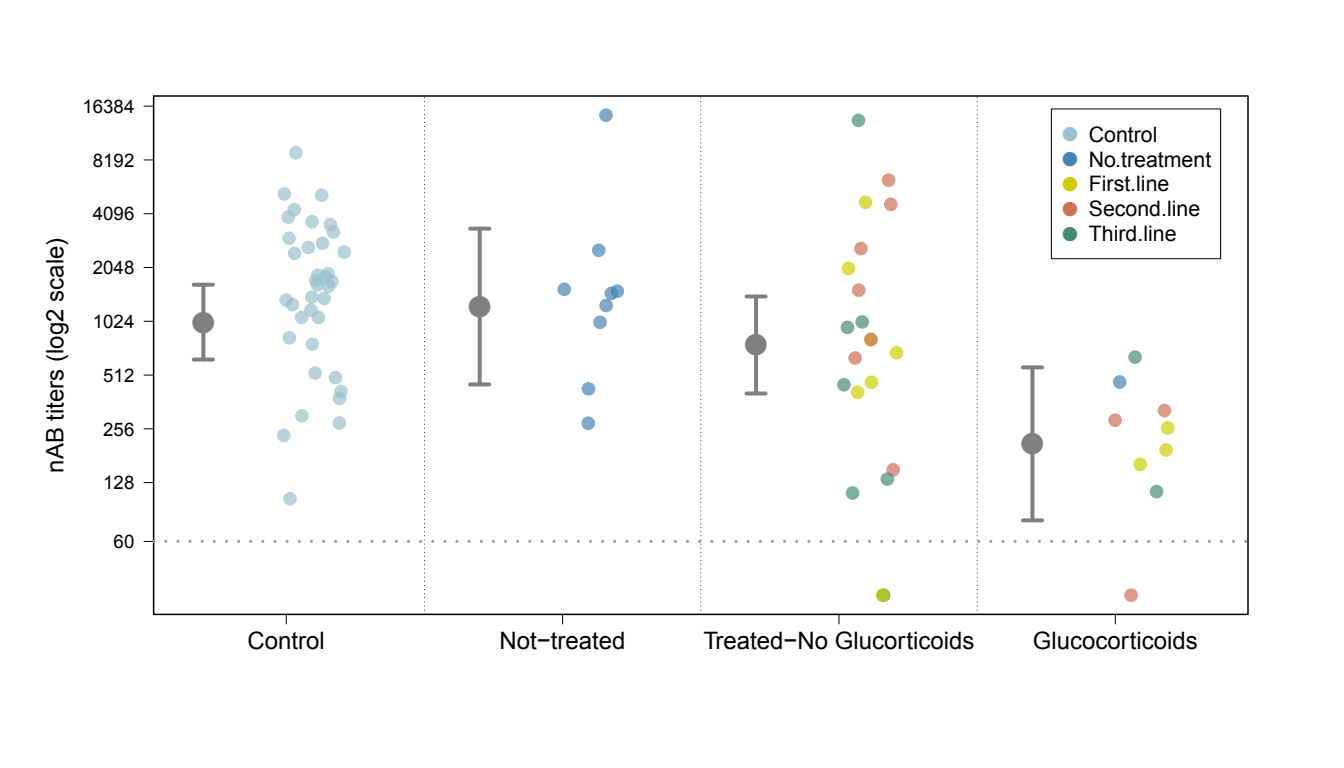


**Figure S1.** Neutralizing antibody (nAB) titre after SARS-CoV-2 vaccination in Healthy individuals (Controls) and Systemic Lupus Erythematosus (SLE) patients by treatment group and use of Glucocorticoids. Diamond-shaped symbols and their associated segments represent adjusted means and 95% confidence intervals of antibody titres. Estimations are derived from a linear model in which sex, age, time from vaccination, vaccine type and corticoids therapy were included as covariates for statistical control. Antibody titres were log2-transformed in order to fit the assumptions of the model and are represented in log2-scale. The horizontal dotted line indicates the detection threshold for the determinations (60). The estimation of the decrease in nAB titres in Lupus patients associated to Glucocorticoids therapy is 75% (Fold-Change = 0.25, 95% Confidence Interval = 0.10-0.63, p-value = 0.0037).


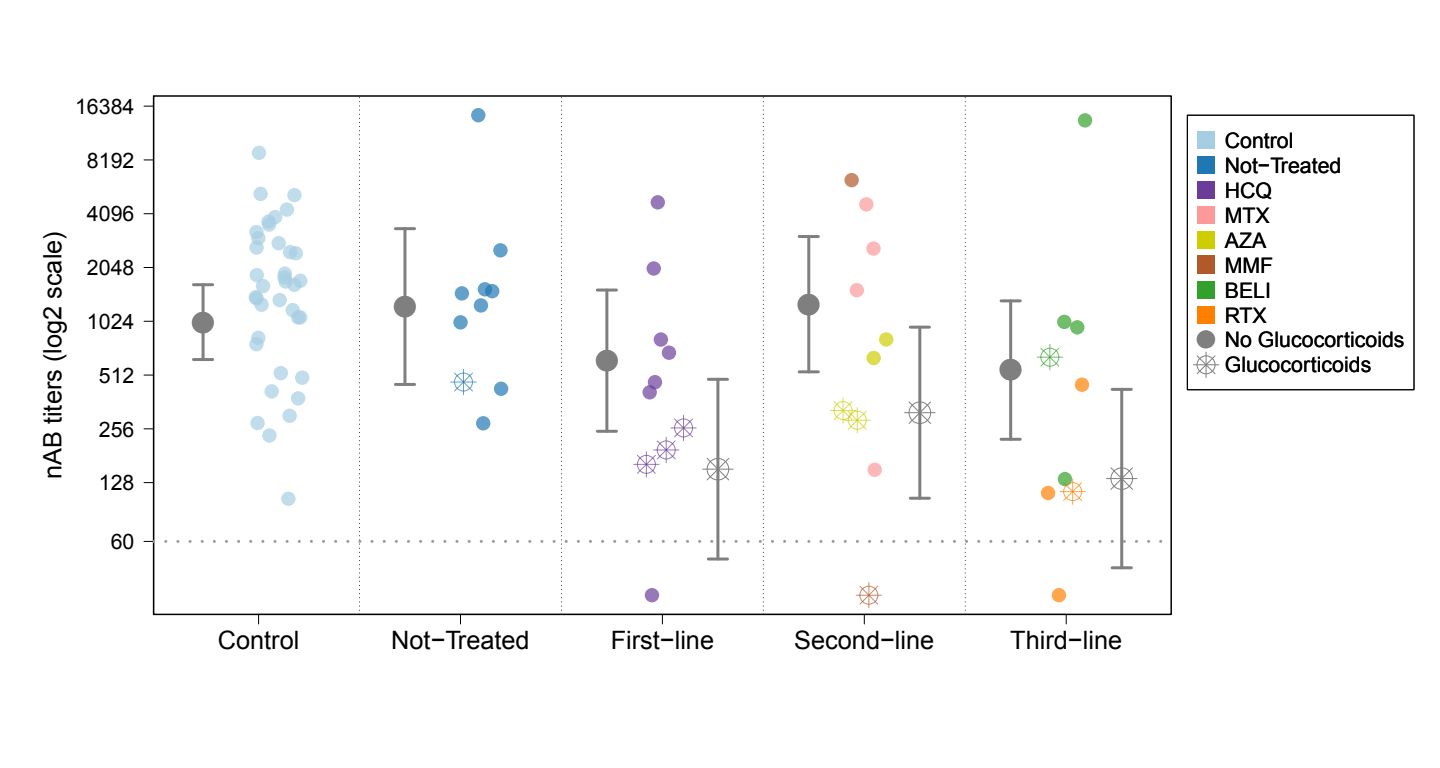


**Figure S2.** Neutralizing antibody (nAB) levels after SARS-CoV-2 vaccination in healthy individuals (Controls) and Systemic Lupus Erythematosus (SLE) patients under different therapy regimens. Circles represent samples of patients not treated with Glucocorticoids, while star-shaped points correspond to patients not under Glucocorticoids regimen. Data points are coloured according to their disease status (Healthy Control or SLE patient) and treatment. Gray points and their associated segments represent adjusted means and 95% confidence intervals of nAB titers. Estimations are derived from a linear model in which sex, age, time from vaccination, vaccine type and Glucocorticoids therapy were included as covariates for statistical control. nAB titers were log2-transformed in order ot fit the assumptions of the model and are represented in log2-scale. The horizontal dotted line indicates the detection threshold for the determinations (60). The estimation of the decrease in antibody titres in SLE patients associated to Glucocorticoids therapy is 75% (Fold-Change = 0.25, 95% Confidence Interval = 0.10-0.63, p-value = 0.0037). **HCQ**: Hydroxychloroquine; **MTX**: Methotrexate; **AZA**: Azathioprine; **MMF**: Mycophenolate Mofetil; **BELI**: Belimumab; **RTX**: Rituximab.
